# Raman enhancement of rhodamine adsorbed on Ag nanoparticles self-assembled into nanowire-like arrays

**DOI:** 10.1186/1556-276X-6-629

**Published:** 2011-12-14

**Authors:** Marianthi Panagopoulou, Nikolaos Pantiskos, Panos Photopoulos, Jun Tang, Dimitris Tsoukalas, Yannis S Raptis

**Affiliations:** 1 Physics Department, School of Applied Mathematical and Physical Sciences, National Technical University of Athens, Zografou Campus, Zografou, Athens 157 80, Greece; 2 Department of Electronics, Technological Educational Institute of Athens, 12210 Aegaleo, Greece; 3 North University of China, Taiyuan, Shanxi, People's Republic of China; 4 Institute of Microelectronics, NCSR "Demokritos", GR 15310 Ag. Paraskevi Greece

**Keywords:** SERS, self-aligned silver nanoparticles, R6G, Raman spectra, nanotechnology (design-applications)

## Abstract

This work reports on Raman scattering of rhodamine (R6G) molecules absorbed on either randomly distributed or grating-like arrays of approximately 8-nm Ag nanoparticles developed by inert gas aggregation. Optimal growth and surface-enhanced Raman scattering (SERS) parameters have been obtained for the randomly distributed nanoparticles, while effects related to the aging of the silver nanoparticles were studied. Grating-like arrays of nanoparticles have been fabricated using line arrays templates formed either by fracture-induced structuring or by standard lithographic techniques. Grating structures fabricated by both methods exhibit an enhancement of the SERS signal, in comparison to the corresponding signal from randomly distributed Ag nanoparticles, as well as a preferential enhancement in the areas of the sharp features, and a dependence on the polarization direction of the incident exciting laser beam, with respect to the orientation of the gratings structuring. The observed spectroscopic features are consistent with a line-arrangement of hot-spots due to the self- alignment of metallic nanoparticles, induced by the grating-like templates.

## Introduction

The effect of the surface-enhanced Raman scattering (SERS) has been observed, since 1974, in (organic) molecules being in close contact with properly structured metallic surfaces, especially nanosized metallic particles. According to this effect, an enhancement, by many orders of magnitude, of the Raman signal from organic molecules was observed, when these molecules were attached to metallic (usually Ag, Au, but also Ni, Co, etc) nanoparticles. An intensive research activity has been carried out, in the following decade, concerning the mechanisms which are responsible for the strong enhancement of the scattering intensity, with respect to the signal of the conventional Raman scattering, with two models, namely, the electromagnetic enhancement and the electrochemical enhancement, proposed as the dominant ones (for a review of the related activities in the decade 1974 to 1984, see [1,2]; see also [3].

Taking into account that in the SERS effect, in addition to the active molecule, which is the actual scattering center (e.g., pyridine, rhodamine, etc.), the most important role is played by the characteristics of the metallic surface, i.e. the metal itself (with prevailing, Ag and Au), and the nanostructure of the metallic surface (several geometries, with typical sizes from several nanometers up to a few tens of nanometers); the corresponding interest has been revitalized the last decade (2000 to 2010), due to the fast development of the nanotechnology and the related structures and devices. The shape and the dimensions of the metallic nanoparticles are strongly affecting the intensity enhancement, since those parameters have an important influence in the spectrum of the surface plasmons [4], while especially interesting are the two-dimensional arrays of nanopillars or nanowires, where SERS effect could be observed even for metals, such as Ni and Co, which were not considered as effective SERS-active [5] materials. According to theoretical studies, the characteristics of the plasmon resonances are correlated with the cross-section of the nanowires [6], with obvious selective scattering applications in the nanosystems [7]. The arrangement of the nanowires in periodic arrays (as a result of either a self- or an induced organization) exhibits related polarization characteristics in the SERS effect [8], quite similar to polarization effects obtained through random parallel nanostructures in gold thin films [9].

The research in this field has been revived during the last years, from both aspects of view, the basic physics (plasmonic properties of random and periodic metallic nanostructures) and chemistry, as well as the potential of new technological tools in the area of chemical sensors. This generation of sensors, which is based on plasmonic excitation effects, is very promising in the fields of chemistry, biochemistry, and biomedical research.

### Experimental details

Two sets of SERS-active samples are studied by microscopic and spectroscopic techniques. The first set, consisting of randomly distributed Ag nanoparticles, is used to obtain the optimal growth and SERS parameters. It is also used to check the influence, to the SERS efficiency, of the different substrates and of the time elapsed since the dying of the nanoparticles. The second set consists of silver nanoparticles evaporated over grating-like structured surfaces. Self-alignment effects of the nanoparticles, due to the structure of the surfaces, are monitored by scanning electron microscopy (SEM) and atomic force microscopy (AFM) techniques, while its influence on the Raman enhancement is studied by micro-Raman scanning and polarization-dependent SERS measurements.

### Growth: preparation

**Figure 1:**
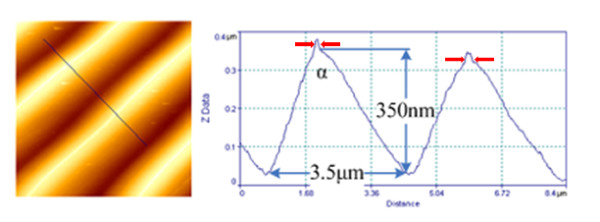
**AFM top view and cross-section profile of the structures fabricated by the FIS method**. Nanoparticle deposition, results in nanoparticle accumulation, shown by the small peak at the top of the structures, (pointing-arrows).

**Figure 2:**
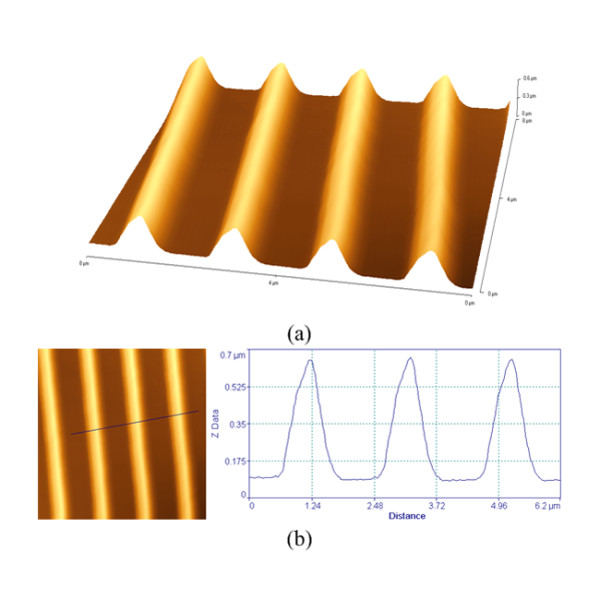
**AFM images of the oxide arrays fabricated by optical lithography and wet etching**. **(a) **Oblique view. **(b) **Top view and cross-section profile (along the black trace) before the nanoparticle deposition.

**Figure 3:**
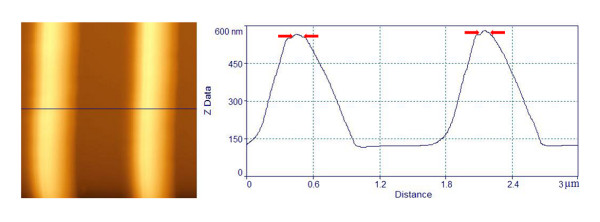
**AFM top view and cross-section profile (along the black line), of the lithographically prepared structure**. (After nanoparticle deposition) A small peak indicated by the pointing-arrows, is observed at the top of the structure due to self-assembled NPs.

Silver nanoparticles, with typical sizes of 8 to 18 nm, were deposited through an inert gas aggregation method [10], in different substrates, like Si, SiO_2_, quartz, and epoxy resin. AFM images of the randomly distributed Ag nanoparticles are shown in an additional file [see Additional file 1]. Grating-like arrays of nanoparticles have been fabricated by depositing Ag nanoparticles on substrates properly configured before the deposition, either by standard lithographic techniques, or by a technique based on fracture-induced structuring [11] (FIS). The gratings prepared by the FIS method were made on silicon and quartz substrates. For this approach, AZ5214 type of photoresist was used, spin coated at 5,000 to 7,000 rpm, and baked at 60°C for 30 to 40 min, under continuous pressure between the two similar substrates, to assure better adhesion. Sudden detachment of the two substrates results in the spontaneous formation of the fracture-induced patterning, which is used in the next step, as the substrate for the AgNP deposition. Using AFM and FESEM it is demonstrated that the Ag nanoparticles are preferentially deposited at the peaks of the lines forming self-assembled nanowires [12]. Representative AFM images illustrating the nanowire formation on the peaks are shown in Figure 1. FIS method is very simple to apply but it suffers from wafer-scale manufacturability. For that reason, we also propose and demonstrate the preparation of triangular cross-section gratings at wafer scale made using conventional processing. The lithographically prepared gratings were constructed on Si substrate following a wet thermal oxidation that resulted to the formation of 1 μm oxide. Then optical lithography and 0.5-μm wet etching in diluted HF were used to create parallel oxide trenches of triangular cross-section. In an additional file [see Additional file 2] we show the process sequence developed to form these oxide arrays as demonstrated by AFM measurements shown in Figure 2. In Figure 3, we show AFM measurements of the same gratings after nanoparticle deposition, where a small peak is shown on top of the triangular structure.

**Figure 4:**
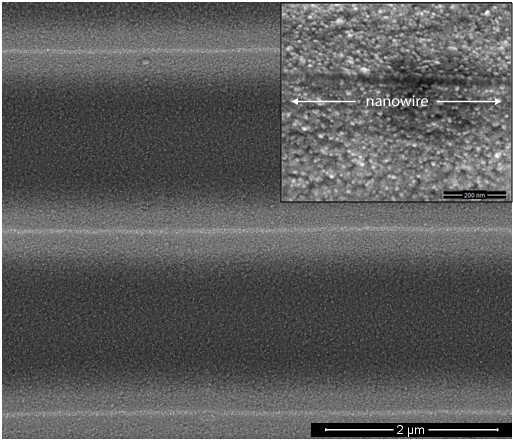
**SEM image**. The edge-driven preferential alignment of silver nanoparticles on the edges of lithographically prepared stripes. Scale-length: 2 μm, (200 nm, in the inset).

Concerning the arrangement of the nanoparticles in lithographically formed stripe templates, it is shown [12] that they are preferentially aligned along the edge lines of the stripes, through an electrostatic self-assembling. A SEM image of this edge-driven preferential alignment is presented in Figure 4. The silver nanoparticles, accumulated on top of the grating-like structures, are preferentially aligned in such a way as to have much more contact-points along, rather than perpendicular, to the axis of the structure. Due to this arrangement, we have a denser concentration of hot-spot points (confirmed by the stronger enhancement of the SERS signal from these structures, as well as by the mapping across the structure), with a preferential orientation of them along the axis of the structure (confirmed by the polarization measurements of the SERS signal); both confirmations are presented in the next paragraph. This configuration of the predominant hot-spot orientation is similar to a nanowire structure.

### Spectroscopy

The study of the silver nanoparticle systems, by SERS spectroscopy, was carried out in macro- and micro-Raman configurations. The samples to be characterized by SERS spectroscopy were immersed, for 12 h, in methanol (or water) solution of rhodamine (R6G), with molarities of the order of magnitude of 10^-^4 M, and then dried by free evaporation of the solvent, for a few minutes. The Raman measurements were taken in ambient conditions. We have tried different wavelengths and have obtained optimum SERS spectra with the 514.5-nm Ar^+ ^laser line. For the macro-Raman measurements, a SPEX 1403 double monochromator with standard photon-counting system was used. For this series of measurements, we have used 20-mW excitation beam power, focused by 75-mm focal-length lens, either cylindrical or spherical, depending on the specific measurements. In this series of measurements, a fluctuation of the SERS intensity was observed, immediately after illumination with the laser beam. All the measurements were taken after a stabilization interval of half an hour after illumination, in order to overcome this effect, known as photobleaching. For the micro-Raman measurements, a JY T64000 triple monochromator, with optical microscope of magnification up to ×100, and a liquid nitrogen-cooled CCD detector was used, equipped with motorized stepping drive motors for the scanning of the grating-like structures. For this series of measurements, an excitation beam power of 0.01 to 0.05 mW, was used, in order to minimize heating and photobleaching effects.

## Results and discussion

### Randomly distributed Ag nanoparticles

**Figure 5:**
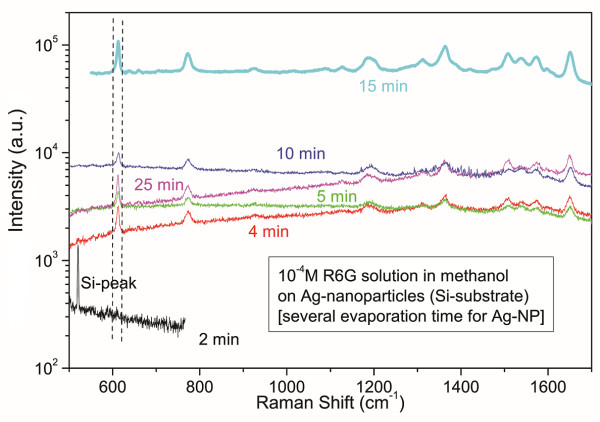
**R6G-SERS spectra from randomly distributed AgNPs, for several deposition times**.

Randomly distributed Ag nanoparticles were studied as a function of deposition time, in order to obtain the optimum evaporation condition, concerning the maximization of the enhancement factor. In Figure 5, the SERS spectra for different nanoparticle deposition times are shown. The SERS R6G spectra exhibit the enhancement of the 612 cm^-1 ^mode, which is characteristic of the silver-nanoparticle-mediated SERS, in contrary to other cases like the gold-nanoparticle-decorated InP nanowires [13] where the enhancement is manifested by the high frequency part (1,300 to 1,700 cm^-1^).

**Figure 6:**
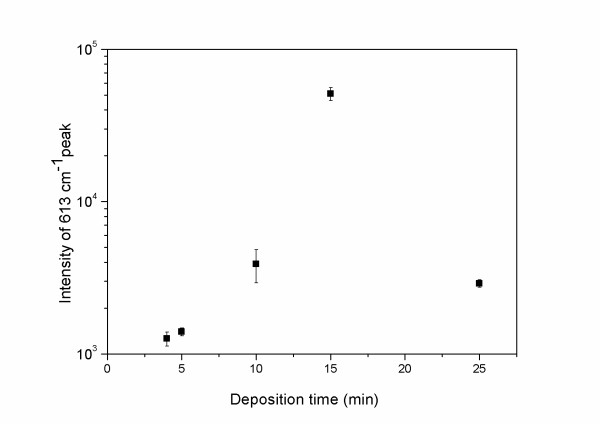
**Deposition-time dependence of SERS intensity from randomly distributed AgNPs, (after subtraction of luminescence background)**.

An increasing SERS signal is observed with increasing deposition time, tending however to a saturation level, as it is shown in Figure 6. This dependence is attributed to the increasing surface density of the Ag nanoparticles, in the initial stage. At a deposition time of 20 to 30 min, the substrate surface becomes fully covered, and any further increase of the deposition time does not increase any more the number of the surface nanoparticles on which SERS-active (R6G) molecules can be anchored. Moreover, it results in the formation of nanoparticle aggregations in larger size clusters, which act as less-efficient SERS-mediating particles (due to lower curvature), leading to a saturation of the enhancement.

**Figure 7:**
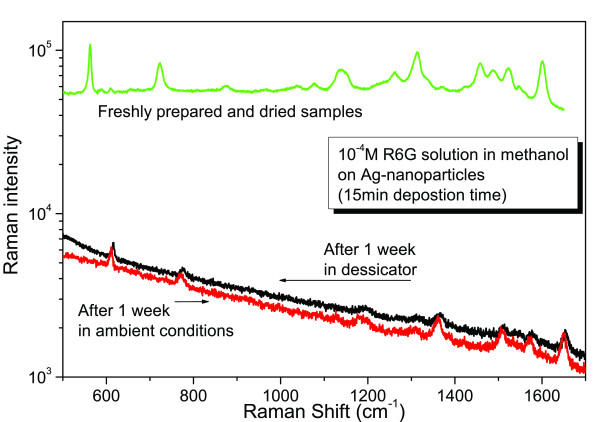
**R6G-SERS spectra, from randomly distributed AgNPs, showing aging effects, and dependence on the storing environment**.

The exposure to the atmosphere of the Ag nanoparticles, immersed in R6G-solution, incites a gradual degradation of the SERS effect. This degradation is more intense when the exposure to the atmosphere is combined with the illumination of the sample by the excitation laser beam. These aging effects are shown in Figure 7, where freshly prepared samples are compared with samples after 1 week in ambient conditions and in a desiccator. Such effects of silver tarnishing, by time, are also known in the literature. More detailed work is in progress, in order to systematically test the influence of the parameters like the duration and the environment of exposure, as well as the illumination of the samples under vacuum, for the SERS measurements. In addition, preliminary attempts related to the protective covering of the Ag nanoparticles with a passivation silicon oxide layer have faced the problem of a diminishing SERS signal because, probably, of the weak coupling of R6G molecules with the surface plasmon excitations of the AgNPs, due to the passivation layer. Therefore, all the consequent measurements were carried out following the same procedure, in order to minimize the aging-related fluctuations in the SERS intensity.

### Periodic arrays

**Figure 8:**
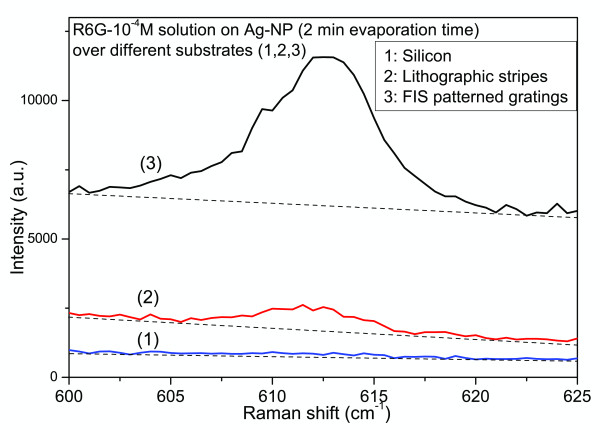
**SERS from different structures, and a comparison of the corresponding scattering intensities**. Above the R6G luminescent background.

Periodic arrays of Ag nanoparticles arranged in parallel lines, were studied by SERS techniques. Using different fabrication methods (lithographic or FIS) and different substrates (Si or quartz), we were able to compare the SERS efficiency from the corresponding combinations. Two types of SERS (R6G) measurements were carried out in the structured nanoparticles. With macro-Raman measurements, we were able to compare the average behavior of differently structured and non-structured areas (and even nanoparticle-free substrates). In order to show clearly the further increase of the SERS (R6G) signal, observed in the case of AgNPs evaporated over structured substrate, the comparison of the SERS intensities is carried out regarding samples with low evaporation times (i.e., 2 to 4 min) for the silver nanoparticles, since these evaporation times correspond to the lower SERS-detection limit, in the case of the unstructured substrates. For this measurements, a cylindrical lens (focal length of 75 mm) was used, with an elliptical spot size of typical semi-axis dimensions 1,000 × 25 μm, resulting in a sampling area of ≈80 × 10^3 ^μm^2 ^= 0.08 mm^2^. The increase of the SERS signal, on the structured nanoparticles areas, is manifested more clearly in samples with low deposition time (e.g., 2 min) like the ones shown in Figure 8, where the FIS-patterned nanoparticles (spectrum 3) present an enormous enhancement with respect to the nanoparticles over lithographic stripes (spectrum 2), $\left(I_{\text{FIS}}\;/)\;I_{\text{Lith}}\approx 10\right)$, and, especially, to the randomly distributed nanoparticles over silicon (spectrum 1), $\left(I_{\text{FIS}}\;/)\;I_{\text{Random}}\approx 10^{2}\right)$ (the dashed lines are indicative of the photoluminescence background of R6G). These factors have to be considered as further amplification of the enhancement coefficient, obtained in the case of the randomly distributed AgNPs, which varies by two orders of magnitude, depending on the deposition time (see Figures 5 and 6), estimated, by independent measurements, to be of the order of 10^4^, at least.

**Figure 9:**
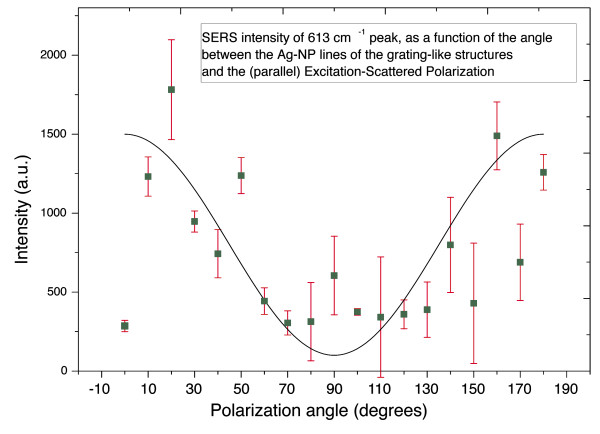
**Polarization dependence of SERS signal, from grating-like structure**. Angle is measured with respect to the axis of the gratings, along which the nanoparticles are preferentially aligned, see text.

In the same macro-Raman configuration, polarization measurements have been carried out. The polarization of the excitation and the scattered light were kept constant, in an exact back scattering geometry (in order to eliminate Brewster angle effects, for the incident radiation, and polarization dependence of the spectrometer efficiency, for the scattered radiation), while the sample was in a goniometric table rotating about the incident-scattering axis. A spherical lens of the same focal length (75 mm) was used, instead of the cylindrical one, in order to minimize effects related to the percentage change of the structured area within the illuminated-sampling area, during the sample rotation. The polarization dependence of the scattered light is shown in Figure 9, where a square-sine curve is also presented to assist the eye. The intensity variation exhibits a maximum when the polarization is parallel to the axis of the aligned nanoparticles, (0° and 180°, in Figure 9), and a minimum when it is perpendicular to these lines (90°). The deviation of the 90° point, of Figure 9, from the minimum value is due to statistical reasons. The specific data points correspond to macro-Raman measurement, with a rotating goniometric stage (for reasons described above). It is very probable, therefore, during the rotation of the sample to excite region of the grating-like structures with slightly different nanoparticle density or rhodamine concentration. The overall dependence is in accordance with the literature where, in similar systems, it is found that the SERS signal is stronger when the excitation polarization is perpendicular to the interface between nanocubes and nanowires [14], or parallel to the axis of nanoparticle pairs [15]. Concerning our system, these observations can be explained by a hot-spot model. According to this model, when a rhodamine molecule lies between two close laying AgNPs, the electromagnetic mechanism of enhancement is expected to contribute stronger when the excitation polarization is parallel to the axis connecting the centers of the two metallic nanoparticles, due to the enhancement of the electromagnetic field, between the adjoining nanoparticles.

**Figure 10:**
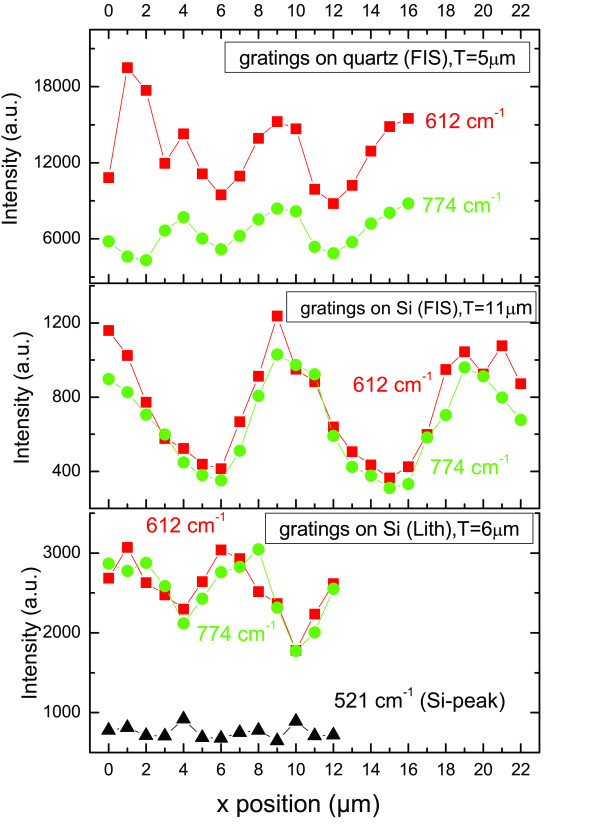
**Micro-Raman scanning of AgNP-decorated grating-like structures**. Showing a spatial modulation of the SERS intensity with the periodicity of the structures. Fabrication parameters are given in the figure insets, while the AgNP deposition time is 4 min for all the structures. Zero value of the horizontal "x position" scale corresponds (within the approximately 1-μm resolution of the micro-Raman system) to the peaks of the grating structures.

Finally, the grating-like structured nanoparticles were examined by micro-Raman technique. Samples of several grating-periodicities and deposition times have been measured. The samples were scanned perpendicularly to the grating lines with different scanning steps (depending on the periodicity of each structure) and a spatial resolution of approximately 1 μm. During the scanning, a spectral range of 500 to 900 cm^-1 ^was recorded, and the intensity of two characteristic Rhodamine peaks (612 and 774 cm^-1^) is monitored. In Figure 10, the intensity of those two peaks is plotted as a function of the position, for three samples of different structural characteristics but with the same deposition time, of 4 min, for the silver nanoparticles. Two of them are fabricated by the FIS method in different substrates (quartz and Si), while the third one is made on Si, by lithographic method. The intensity variation exhibits a, more or less, clear periodicity similar to the periodicity of the structure, which is indicated by the *T *values; (see the inset labels of Figure 10). It is worth to note that the absolute SERS signal, as far as it concerns the FIS samples, is by one order of magnitude stronger in the case of low pattern periodicity (i.e., 5 μm), in comparison to the absolute signal of a doubled periodicity (11 μm). The maxima are unambiguously, located in the peaks of the grating structures, as it is visually confirmed through the microscope of the micro-Raman system. In the case of the higher periodicity (≥ 10 μm), a more clearly defined depth of modulation, as a function of the position, is observed, because, (with such a periodicity), we have enough spatial resolution (through of the approximately 1-μm-spot size of the micro-Raman detection), to clearly reveal the further enhancement of the SERS signal induced by the edge-driven alignment of the nanoparticles accumulated on the peaks of the grating structures. However, the stronger enhancement observed in low periodicities might be of importance from the applications point of view.

## Conclusions

In conclusion, we have presented SERS-based investigation on randomly distributed and periodically self-arranged metallic nanoparticles. Optimization parameters obtained through the randomly distributed NPs, were taken into account in the study of the periodic structures. These studies confirm, through polarization measurements and micro-Raman scanning, the signal enhancement from the nanoparticles which are aligned in a form of nanowires, and prove that the synergetic combination of the two spontaneous organization processes (FIS patterning and nanoparticle self-alignment) can lead to further SERS enhancement with physically interesting aspects and potentially promising technological consequences.

## Competing interests

The authors declare that they have no competing interests.

## Authors' contributions

MP has prepared the FIS and lithographic substrates for Ag-NPs deposition, collected and analyzed the SERS data on periodic structures. NP has participated in the randomly distributed Ag-NPs deposition, SERS acquisition and analysis. PP has participated in the design and realization of the Ag-NPs deposition for all the sample sequences. JT has participated in the acquisition and analysis of the AFM images of the Ag-NPs on the periodic structures. DT has participated in the conception and design of the different sequences of the structured substrates and Ag-NPs deposition. YSR has participated in the conception, design and realization of the SERS measurements of random and structured substrates. DT and YSR conceived and designed the study. All authors have been involved in drafting and revising the manuscript and have given the final approval of the version to be published.

## Supplementary Material

Additional file 1 **AFM image of randomly distributed silver nanoparticles**. We have initially studied the deposition conditions to obtain controlled density and size distributions of the nanoparticles. Using Transmission Electron Microscopy (TEM), we have found that by changing the deposition conditions like substrate temperature, deposition time, and DC power, we can control the surface density of the nanoparticles as well as their nominal size which are up to 1,012 cm^-2 ^and 2 to 14 nm, respectively. In the AFM figure above, we demonstrate the results after 4 min deposition of silver nanoparticles of 8 nm initial size and final average size of 25 nm. (http://www.nanoscalereslett.com/imedia/9775342116065051/supp1.pdf). Click here for file

Additional file 2 **Schematic of the lithographic fabrication procedure for the SiO_2 _line arrays used as structured substrate for the self-assembled AgNPs**. We show the process sequence to obtain silicon oxide lines with triangular cross-section in an attempt to fabricate a completely manufacturable template at wafer scale. Fabrication process of the SiO2 line arrays with peak structures: **(a) **Silicon wafers with 1-μm thickness of thermal SiO2 were prepared and a photoresist layer was spin coated on the surface. **(b) **By means of optical lithography, the photoresist was patterned into arrays of lines with 1-μm periodicity. **(c) **The wafer was etched isotropically in diluted HF. **(d) **Photoresist was finally removed in acetone. (http://www.nanoscalereslett.com/imedia/1316397287556978/supp2.pdf). Click here for file

## References

[B1] Otto A Cardona M, Guntherodt G Surface-Enhanced Raman Scattering: "Classical" and "Chemical" Origins Light Scattering in solids IV: topics in applied physics 1984 Chapter 6 New York: Springer 289 418

[B2] Arya K Zeyher R Cardona M, Guntherodt G Theory of Surface-Enhanced Raman Scattering Light Scattering in solids IV: topics in applied physics 1984 Chapter 6 New York: Springer 419 462

[B3] Moskovits M Surface-enhanced spectroscopy Rev Mod Phys 1985 57 783 10.1103/RevModPhys.57.783

[B4] Mock JJ Barbic M Smith DR Schultz DA Schultz S Shape effects in plasmon resonance of individual colloidal silver nanoparticles J Chem Phys 2002 116 6755 10.1063/1.1462610

[B5] Yao JL Pan GP Xue KH Wu DY Ren B Sun DM Tang J Xu X Tian ZQ A complementary study of surface-enhanced Raman scattering and metal nanorod arrays Pure Appl Chem 2000 72 221 10.1351/pac200072010221

[B6] Kottmann JP Martin OJF Smith DR Schultz S Non-regularly shaped plasmon resonant nanoparticle as localized light source for near-field microscopy Phys Rev B 2001 64 235402 10.1046/j.1365-2818.2001.00866.x 11298871

[B7] Tao A Kim F Hess C Goldberger J He R Sun Y Xia Y Yang P Langmuir-Blodgett silver nanowire monolayers for molecular sensing using surface-enhanced Raman spectroscopy Nano Letters 2003 3 1229 10.1021/nl0344209

[B8] Jeong DH Zhang YX Moskovits M Polarization-Dependent Surface-Enhanced Raman Scattering from a Silver-Nanoparticle-Decorated Single Silver Nanowire J Phys Chem B 2004 108 12724 10.1021/jp037973g 18767889

[B9] Brolo AG Arctander E Addison CJ Strong Polarized Enhanced-Raman Scattering via Optical Tunneling through Random Parallel Nanostructures in Au Thin Films J Phys Chem B 2005 109 401 10.1021/jp046045u 16851029

[B10] Tang J Verrelli E Tsoukalas D Assembly of charged nanoparticles using self-electrodynamic focusing Nanotechnology 2009 20 36 365605 10.1088/0957-4484/20/36/365605 19687554

[B11] Pease LF Deshpande P Wang Y Russel WB Chou SY Self-formation of sub-60-nm half-pitch gratings with large areas through fracturing Nature Nanotechnology 2007 2 545 548 10.1038/nnano.2007.264 18654365

[B12] Tang J Verrelli E Giannakopoulos K Tsoukalas D Electrostatic self-assembly of nanoparticles into ordered nanowire arrays J Mater Res 2011 26 209 214 10.1557/jmr.2010.16

[B13] Chen J Mårtensson T Dick KA Deppert K Xu HQ Samuelson L Xu H Surface-enhanced Raman scattering of rhodamine 6G on gold nanoparticles deposited 3-dimensionally on nanowire arrays Nanotechnology 2008 19 275712 10.1088/0957-4484/19/27/275712 21828724

[B14] Camargo PHC Cobley CM Rycenga M Xia Y Measuring the SERS enhancement factors of hot spots formed between an individual Ag nanowire and a single Ag nanocube Nanotechnology 2009 20 434020 10.1088/0957-4484/20/43/434020 19801754

[B15] Theiss J Pavaskar P Echternach PM Muller RE Cronin SB Plasmonic nanoparticle arrays with nanometer separation for high-performance SERS substrates Nano Letters 2010 10 2749 10.1021/nl904170g 20698586

