# Supplementary material for: Raman enhancement of rhodamine adsorbed on Ag nanoparticles self-assembled into nanowire-like arrays
Source: Nanoscale Res Lett. 2011 Dec 14;6(1):629. doi: 10.1186/1556-276X-6-629 (PMC3278473; doi:10.1186/1556-276X-6-629)
Supplement: Additional file 1 — AFM image of randomly distributed silver nanoparticles. We have initially studied the deposition conditions to obtain controlled density and size distributions of the nanoparticles. Using Transmission Electron Microscopy (TEM), we have found that by changing the deposition conditions like substrate temperature, deposition time, and DC power, we can control the surface density of the nanoparticles as well as their nominal size which are up to 1,012 cm-2 and 2 to 14 nm, respectively. In the AFM figure above, we demonstrate the results after 4 min deposition of silver nanoparticles of 8 nm initial size and final average size of 25 nm. (http://www.nanoscalereslett.com/imedia/9775342116065051/supp1.pdf). [file 1556-276X-6-629-S1.PDF]

In the following figure we demonstrate the randomly distributed Ag-nanoparticles

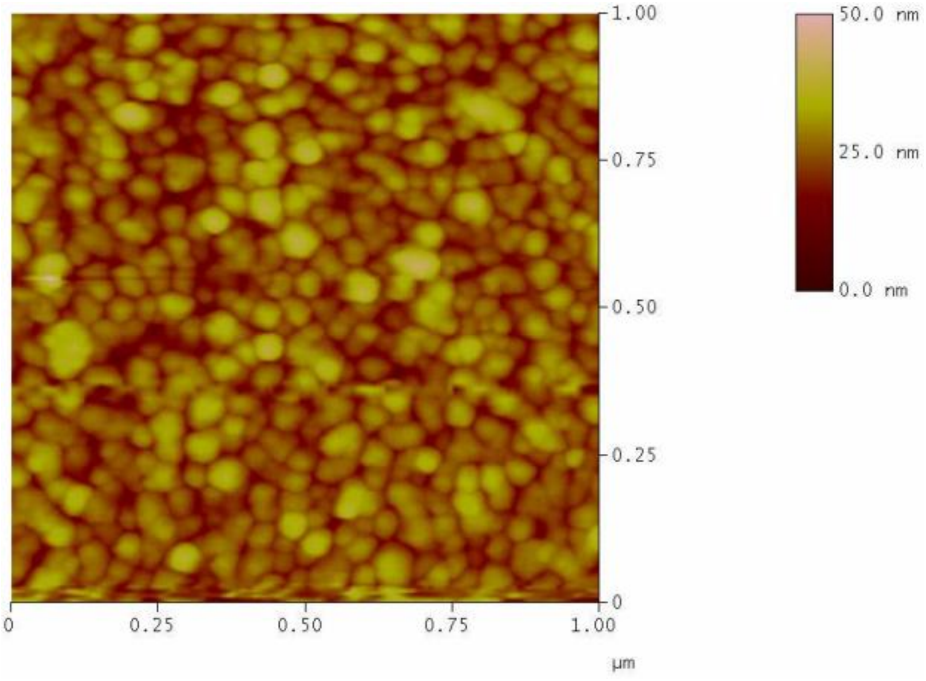

Figure

We have initially studied the deposition conditions to obtain controlled density and size distributions of the nanoparticles. Using Transmission Electron Microscopy (TEM), we have found that by changing the deposition conditions like substrate temperature, deposition time and DC power, we can control the surface density of the nanoparticles as well as their nominal size which are up to 10^{12} cm^{-2} and 2 nm-14 nm respectively. In the AFM figure above, we demonstrate the results after 4 min deposition of silver nanoparticles of 8nm initial size and final average size of 25 nm.
