# Supplementary material for: Raman enhancement of rhodamine adsorbed on Ag nanoparticles self-assembled into nanowire-like arrays
Source: Nanoscale Res Lett. 2011 Dec 14;6(1):629. doi: 10.1186/1556-276X-6-629 (PMC3278473; doi:10.1186/1556-276X-6-629)
Supplement: Additional file 2 — Schematic of the lithographic fabrication procedure for the SiO2 line arrays used as structured substrate for the self-assembled AgNPs. We show the process sequence to obtain silicon oxide lines with triangular cross-section in an attempt to fabricate a completely manufacturable template at wafer scale. Fabrication process of the SiO2 line arrays with peak structures: (a) Silicon wafers with 1-μm thickness of thermal SiO2 were prepared and a photoresist layer was spin coated on the surface. (b) By means of optical lithography, the photoresist was patterned into arrays of lines with 1-μm periodicity. (c) The wafer was etched isotropically in diluted HF. (d) Photoresist was finally removed in acetone. (http://www.nanoscalereslett.com/imedia/1316397287556978/supp2.pdf). [file 1556-276X-6-629-S2.PDF]

In the following figure we show the process sequence to obtain silicon oxide lines with triangular cross section in an attempt to fabricate a completely manufacturable template at wafer scale.

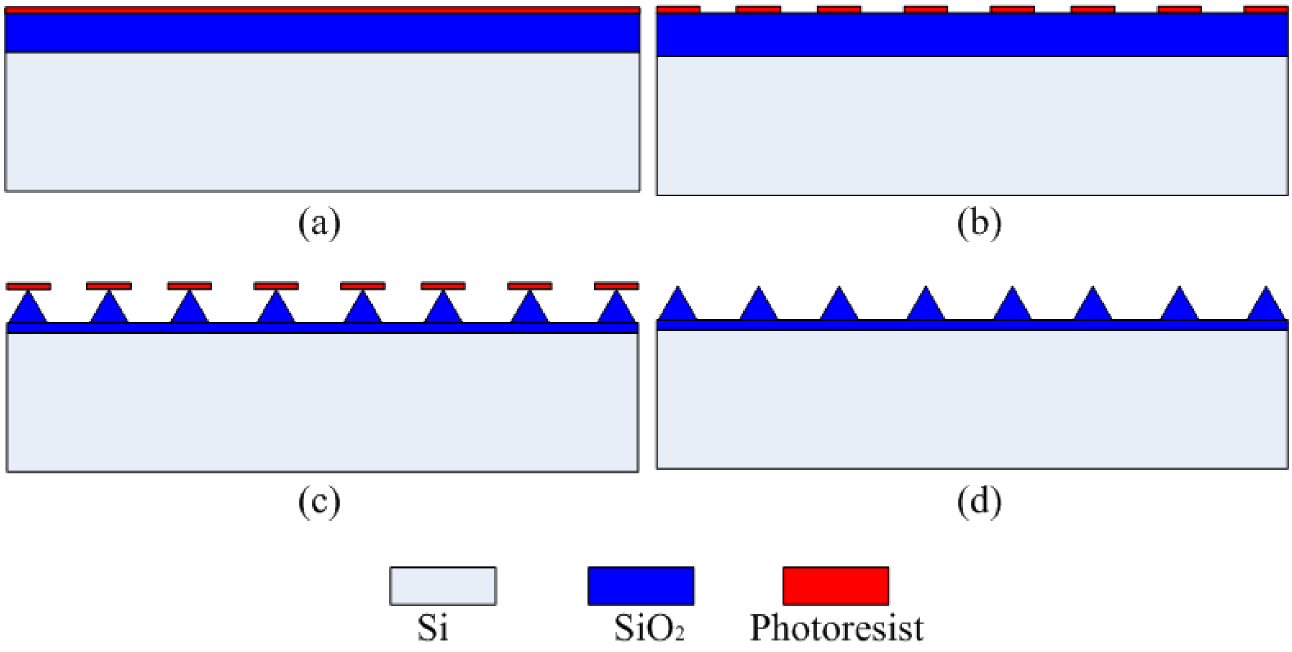

Figure

Fabrication process of the SiO_2 line arrays with peak structures: (a) Silicon wafers with 1 μm thickness of thermal SiO_2 were prepared and a photoresist layer was spin coated on the surface; (b) By means of optical lithography, the photoresist was patterned into arrays of lines with 1μm periodicity; (c) The wafer was etched isotropically in diluted HF; (d) Photoresist was finally removed in acetone.
